# Metagenomic analysis of the microbiome of the upper reproductive tract: combating ovarian cancer through predictive, preventive, and personalized medicine

**DOI:** 10.1007/s13167-022-00286-1

**Published:** 2022-06-23

**Authors:** Xu Qin, Jianglin Zhou, Zizhuo Wang, Chenzhao Feng, Junpeng Fan, Jia Huang, Dianxing Hu, Babak Baban, Shengqi Wang, Ding Ma, Chaoyang Sun, Zhe Zhou, Gang Chen

**Affiliations:** 1 Cancer Biology Research Center, Tongji Hospital, Tongji Medical College, Huazhong University of Science and Technology, Wuhan, China; 2 Department of Stomatology, Tongji Hospital, Tongji Medical College, Huazhong University of Science and Technology, Wuhan, China; 3 Beijing Institute of Microbiology and Epidemiology, Beijing, China; 4 Department of Obstetrics and Gynecology, Tongji Hospital, Tongji Medical College, Huazhong University of Science and Technology, Wuhan, China; 5 Medical College of Georgia, Augusta University, Augusta, GA USA

**Keywords:** Predictive preventive personalized medicine, Ovarian cancer, Microbiome, Metagenomics, Female upper reproductive tract, Platinum therapy, R package, Cowplot package

## Abstract

**Purpose:**

We investigated whether ovarian cancer could alter the genital microbiota in a specific way with clinical values. Furthermore, we proposed how such changes could be envisioned in a paradigm of predictive, preventive, and personalized medicine (PPPM).

**Methods:**

The samples were collected using cotton swabs from the cervical, uterine cavity, fallopian tubes, and ovaries of patients subjected to the surgical procedures for the malignant/benign lesions. All samples were then analyzed by metagenomic shotgun sequencing. The distribution patterns and characteristics of the microbiota in the reproductive tract of subjects were analyzed and were interpreted in relation to the clinical outcomes of the subjects.

**Results:**

While the ovarian cancer was able to alter the genital microbiota, the bacteria were the dominant microorganisms in all samples across all cohorts in the study (median 99%). The microbiota of the upper female reproductive tract were mainly from the cervical, identified by low bacterial biomass and high bacterial diversity. Ovarian cancer had a distinct microbiota signature. The tubal ligation affects its microbial distribution. There were no different species on the surface of platinum-sensitive ovarian tissues compared to samples from platinum-resistant patients.

**Conclusion:**

The ovarian cancer–induced changes in microbiota magnify the potential of microbiota as a biotherapeutic modality in the treatment of ovarian cancer in this study and very likely for several malignancies and other conditions. Our findings demonstrated, for the first time, that microbiota could be dissected and applied in more specific fashion based on a predictive, preventive, and personalized medicine (PPPM) model in the treatment of ovarian cancer. Utilizing microbiota portfolio in a PPPM system in ovarian cancer would provide a unique opportunity to a clinically intelligent and novel approach in the treatment of ovarian cancer as well as several other conditions and malignancies.

**Supplementary Information:**

The online version contains supplementary material available at 10.1007/s13167-022-00286-1.

## Introduction

Ovarian cancer has become the most lethal malignant tumor for women [1, 2]. Given its insidious onset and poor response in advanced stages to various treatments, the development of a vital predictive- and preventive-based strategy is a dire urgency in ovarian cancer [1]. Furthermore, heterogeneity of ovarian cancer necessitates fostering a system in which individuals could be screened for early diagnosis, prevention of advanced stages of the disease, and better clinical outcomes. To accomplish such predictive and preventive standards, it is required that a “personalized” protocol be assembled in which cutting-edge knowledge and technologies in basic and clinical sciences be combined and executed. In this way, the challenge of heterogenicity in ovarian cancer would be converted to a golden opportunity via a “personalize medicine” paradigm. In such scenario, potential vulnerable individuals would be categorized based on their genetic and biologic background and differences; a tailored preventive and/or therapeutic strategy, rather than general one, would be determined for them against ovarian cancer in a personalized manner.

There are few reports on the perspective of using “OMICS” in the context of personalized medicine in ovarian cancer [3]. Genomics and genetic background play a central role in personalization of any care in ovarian cancer. Adopting next generation sequencing (NGS) has been a central piece in exploring novel therapeutic targets in malignancies including ovarian cancer [4]. Besides our late discovery of the close relationship between gene mutation and occurrence of a small portion of ovarian cancers, like BRCA1, BRCA2, Pten, and TP53, the most recent and important understanding about the cause of ovarian cancer is the existence of some pathogenic substances in the lower reproductive tract. These pathogens may play certain cancer-causing effects, which explains why ovarian cancer usually starts at the tail ends of the fallopian tubes [5–8]. Moreover, tubal ligation and hysterectomy lower the risk of ovarian cancer by 20 ~ 30% [9]. These clinical observations partly challenge the inherent knowledge that the ovarian located in an isolated environment lacks active communication with outside for anatomical reasons. This would rationalize exploring a new domain of omics, namely, “Microbiom” which magnifies the dire need for unraveling the pathogenic or beneficial role of microorganisms in ovarian cancer on a personalized fashion. Additionally, embracing such novel niche of microbiome would be highly helpful in preventive and predictive measures regarding ovarian cancer as well as future direction of current research concerning women’s health even in more general manner beyond ovarian cancer.

Microorganisms, including viruses and bacteria, have been suspected of having a role in carcinogenesis for a long time [10]. However, as for the upper reproductive tract, including the ovarian, the microorganism’s existence is difficult to detect, making the analysis of its pathogenetic effect even more challenging. A 2016 clinical study suggested that the local microbiome of endometrial cancer patients disrupted, compared with benign uterine samples [11]. Our previous research also found microbial disorders in ovarian cancer tissues [6]. Most studies have found that from the lower reproductive tract to the upper reproductive tract, the microbial biomass is getting less, but the diversity has become richer [12, 13]. However, there still lacks substantial evidence to link microbial dysbiosis and ovarian tumorigenesis directly. On the other hand, researchers found that certain microbiome could influence the therapeutic efficacy of different cancer treatments, including chemotherapy and immunotherapy, for various tumor types [14, 15]. As for ovarian cancer, it is still unknown whether chemotherapy-resistant and chemotherapy-sensitive patients own specific microbial signatures.

Collectively, in this study, we tested our overarching new concept that ovarian microbiota could be used as a reliable source in a PPPM perspective in fight against ovarian cancer. To evaluate such notion, we determined the distribution pattern of ovarian microorganisms and factors which can affect their composition and phenotypic features through a well-designed controlled and systemic clinical study. Our main intention through this current study was to introduce the powerhouse of ovarian microbiota in the context of PPPM as a novel approach to improve and promote the current preventive and therapeutic modalities in the treatment of ovarian cancer.

## Materials and methods

### Patients

This is a prospective study approved by the Review Board of Tongji Hospital (Tongji Medical College, Huazhong University of Science and Technology Institutional) [16]. The research subjects are divided into two groups of the patients with ovarian cancer (experimental group) and the group of patients with benign lesions (control group). While subjects in both groups underwent bilateral salpingo-oophorectomy, patients with malignant ovarian cancer were subjected to metastasis resection and lymph node dissection according to the stage. All subjects stop taking antibiotics 2 weeks prior to the surgery. No chemotherapy was administered to either group. All patients signed the consent form.

### Clinical sample collection

A data file of personal information for every subject was formed (*n* = 65, 35 experimental group and 30 control group). The data file included the basic clinical information and all follow-up trajectory records. Using cotton swab (CY-98000PS, Huachenyang Co., LTD, Shenzhen, China), samples were taken from the surface of cervical (CCT), uterine cavity (EMT), fallopian tube (FTT), and ovarian (OCT). Samples from physical environment of the surgery room and equipments were also taken using cotton swabs to ensure that careful, standardized, and appropriate standards were rigorously employed during the study. All samples were transported temporarily in liquid nitrogen and then transferred to − 80° freezer for longer term storage.

### Metagenomic shotgun sequencing

All samples were prepared for the metagenomic shotgun sequencing according to previous protocols [6]. Briefly, total genomic DNA was extracted using QIAamp DNA Microbiome Kit (Qiagen, USA). After DNA extraction, 1 µg genomic DNA was randomly fragmented by Covaris, followed by purification by AxyPrep Mag PCR clean-up kit. The fragmented DNA was selected by Agencourt AMPure XP Medium kit to an average size of 200–400 bp. The fragments were end-repaired by End Repair Mix and purified afterward. The repaired DNAs were combined with A-Tailing Mix. Then the Illumina adaptors were ligated to the Adenylate 3’Ends DNA and followed by purification. The products were selected based on the insert size. Several rounds of PCR amplification with PCR Primer Cocktail and PCR Master Mix were performed to enrich the Adapter-ligated DNA fragments. After purification, the library was qualified by the Agilent 2100 bioanalyzer (Agilent, USA) and ABI StepOnePlus Real-time PCR System. Finally, the qualified libraries were sequenced on the Illumina Hiseq platform (BGI-Shenzhen, China).

### Metagenomic quality control and taxonomic profiling

All raw sequenced reads were performed quality-control using fastp tool v0.20.1 [17], whereby the adapters were automatically detected and trimmed, low complexity reads were filtered out, read bases corrected, and the reads with length shorter than 36 bp and poor quality (a phred quality value < 20 for > 40% of the read length) were removed. Next, the post-quality-filtered reads were decontaminated by performing end-to-end Bowtie-2 v2.3.5.1 alignment in “very-sensitive” option against human reference genome (GRCh38), phage phiX174 (NC_001422.1), and vector sequences (UniVec & UniVec_Core Database) to exclude any human/phiX/vector DNA contamination [18]. The unmapped reads were extracted and converted to FASTQ files by samtools v1.10 [19]. The clean reads were assigned to microbial taxa using Kraken2 v2.1.1 with prebuilt k2_pluspf_2021027 reference database and 0.1 confidence [20]. The taxonomic abundances were estimated using Braken tool v2.6.2 from Kraken2 outputs [21]. All reads (and taxa) from mitochondria and chloroplast as well as non-fungi eukaryotes were excluded. Except for alpha-diversity analyses, the microbial taxa that only have less than 0.00001% relative abundance in the total dataset or appeared in less than 5 samples were further filtered out to eliminate potentially artifactual sequences.

### Alpha- and beta-diversity analyses

For taxonomic alpha-diversity, clean reads were rarefied to 6172 reads per sample. The rarefaction depth corresponded to the sample with the lowest count of valid reads. A total of four measures of alpha diversity were calculated using R package “phyloseq” [22]: observed richness (the total number of different taxa in a sample), Shannon diversity index (accounts for both taxonomic richness and evenness in a sample), Inverse Simpson index (an indicator of the richness in a sample with uniform evenness), and Chao1 (the total richness of a sample). Alpha diversity difference between two conditions of patients (B and M) or the comparisons among the four different sample sites were performed with the Wilcoxon-rank sum test and Dunn’s test of multiple comparisons, respectively.

For beta-diversity analyses, the microbial taxa count table was centered-log ratio (clr) transformed as described by Aitchion to better handle comparisons of compositional data [6, 23]. Principal component analysis (PCA) analyses were performed with Euclidean distances of clr-transformed counts using R package “phyloseq” [22]. Differences between microbial communities (sample sites and/or patients’ conditions) were determined by PERMANOVA using the function “adonis2” in the R package “vegan” [24].

### Statistical analysis

To find some microbial taxa that generally varied between adjacent body sites alongside tractus genitalis, we specifically extracted samples from all four sites of the same host and made taxonomic profiling comparisons between adjacent body sites for every subject. To be more precise, for every two adjoining sample sites, FTT vs. OCT, we compared the relative abundance of the taxa between samples from FTT sites and those from OCT sites of the same subject. A taxon was considered as a candidate differential abundant taxon if the absolute value of log2-transformed relative abundance difference >  = 0.5. In addition, if a taxon only appeared in one of the adjacent positions and its relative abundance was not less than a specified threshold (0.05%), it was also considered a candidate differential abundant taxon. For these candidate differentials abundant taxa, we considered a microbial taxon significantly increased (or decreased) only if the taxon was shared at least in 75% of samples in that specific group. The significant taxa were individually subjected to an unpaired Wilcoxon-rank sum test of all models from corresponding sample site and condition to fulfill the statistical analysis.

## Results

### General and demographic analysis

Table 1 summarizes the basic information and demographic data for all subjects. As shown, there were 35 patients in the experimental group, with an average age of 52.7 ± 8.9, including 27 serous cell carcinoma, two mucinous carcinoma, three clear cell carcinoma, and three endometrioid carcinoma patients. For 9 cases, the fallopian tube samples were unavailable due to tumor invasion; for 1 case, the cervix was not taken and the cervical and uterine cavity results were not obtained for another subject. There were thirty benign cases (control group) with an average age of 55.1 ± 11.2 in the control group, twenty of which did not undergo ovariectomy. Among the malignant cases, eleven patients were not ligated, and thirteen were ligated. Platinum resistant was defined as relapse 6 months after 1st line platinum-based chemotherapy [25]. Eight cases were platinum-resistant, and nineteen cases were platinum-sensitive.

**Table 1:** Demographic and clinical characteristics of the subjects

	Ovarian cancer	Noncancerous
Number	35	30
Age, years (mean ± SD)	52.7 ± 8.9	55.1 ± 11.2
Type of tumor (*n*, %)		
SC	27	-
CCC	3	-
EC	3	-
MC	2	-
TNM stage (*n*, %)		
I	2	-
II	3	-
III	29	-
IV	1	-
Platinum resistant (*n*, %)		
Yes	8	-
No	19	-
Other	8	-
Tubal ligation (*n*, %)		
Yes	13	7
No	11	17
Other	11	8

### General microbial distribution in the paired samples of both benign and malignant patients

After quality control and de-host analysis, Kraken2 + PlusPF was used for species annotation followed by the seqkit stat used to perform a basic analysis. The average read sequence length of samples from patients in each state was in the range of 61 to 147.3 bp, and the median was in the range of 103 to 144 bp. The base quality of the read sequence of each state sample was acceptable, and the median Q30 ratio was above 96% (SuppFig. 1A). The reads of samples of different parts could be effectively annotated (SuppFig. 1B and C).

### Specific composition of microorganism analysis

**Fig. 1:**
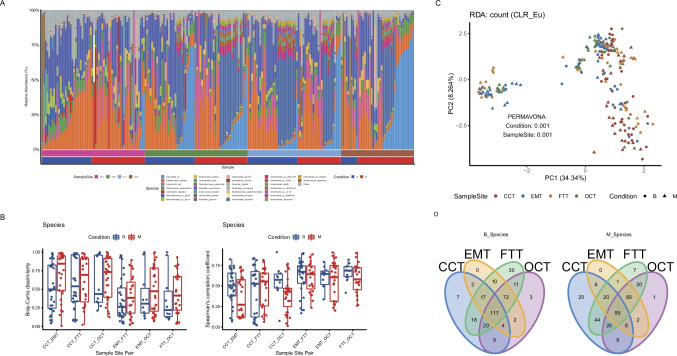
Signature species for each body site. **A** Species-level relative abundance community barplot analysis of metagenomic shotgun sequencing in the cervical (CCT), uterine cavity (EMT), fallopian tube (FTT), and ovarian (OCT) from the benign (B) or malignant (M) lesion patients. **B** Bray-Curtis dissimilarity and Spearman’s correlation coefficient analysis of two adjacent parts at species level. **C** Species-level beta-diversity analysis (Aitchison distance, PCA). There is taxa diversity in different sites in two types of diseases by PERMAVONA analysis (condition: *p* = 0.001, sample site: *p* = 0.001). **D** Veen plot of microbial species among different sample sites

Bacteria had the highest proportion (median of 99%) in all samples compared to the other microorgansms. The ratio of fungi, viruses, eukaryotes, and archaea was relatively small, less than 1% in most samples. At the phylum level, the top 5 relative abundance was Proteobacteria, Firmicutes, Actinobacteria, Bacteroidetes, and Tenericutes (SuppFig. 1E). The Proteobacteria phylum had the highest abundance in all samples. The proportion of Firmicutes in cervical samples was higher than that of the other three parts. The average abundance of *Actinobacteria* bacteria from all malignant patients was higher than that in benign patients, especially in the ovarian cases. At the species level, the OUTs that appear in at least 10% of the samples were conserved. The top 5 were *Pseudomonas tolaasii*, *Klebsiella pneumoniae*, *Salmonella* sp*.*, *Acinetobacter johnsonii*, and *Escherichia coli* (Fig. 1A). Furthermore, species-level alpha-diversity suggested no significant differences in microbial diversity of the cervix, uterine cavity, and fallopian tube except for ovarian tissues (Fig. 1A, SuppFig. 1D).

### Migration analysis

To analyze the migration of microorganisms from the lower reproductive tract to the upper reproductive tract, we conducted a similarity analysis of the adjacent positions of samples from the four parts. At the phylum level, the relative abundance of *Firmicutes* bacteria in both the benign and malignant patients generally decreased from CCT to EMT (SuppFig. 2A and B). The relative abundance of *Ascomycota* bacteria in malignant patients from EMT to FTT was both increased, and the difference was statistically significant (*p* < 0.01) (SuppFig. 2C and D). The relative abundance of *Uroviricota* gate in benign patients from FTT to OCT generally raised, but the difference was not statistically significant (SuppFig. 2E and F). At the level of CCT-EMT, there were apparent differences in the similarity between the two groups (Fig. 1B). There was no significant difference in the parallel of other parts between the two groups; the relative abundance of *Brevundimonas sp. DS20* bacteria in group B patients generally decreased from CCT to EMT. The relative abundance of *Brevundimonas mediterranea*, *Brevundimonas* sp. scallop, *Brevundimonas* sp*.* Bb-A, *Brevundimonas* sp*.* DS20, *Brevundimonas* sp*.* GW460-12–10-14-LB2, *Brevundimonas sp.* SGAir0440, and *Cutibacterium_acness* was reduced from CCT to EMT in the malignant group (SuppFig. 3A and B). *Cutibacterium acnes* and *Komagataella phaffii* generally increased in relative abundance from EMT to FTT in patients in malignant group (SuppFig. 3C and D). The relative abundance of *Cutibacterium acnes* in benign patients from FTT to OCT had an upward trend (*p* > 0.05). The relative abundance of *Brucella intermedia* in the experiment group from FTT to OCT decreased, which had a statistical significant.

For the sample diversity, the beta diversity analysis indicated that there were significant differences in the microbial composition of patients in different groups (*p* <  = 0.001) (Fig. 1C). For presentation purposes, we performed a Venn diagram analysis. The results suggested that the out shared by four parts accounted for the majority, indicating that the lower reproductive tract was the primary source of microorganisms for the upper reproductive tract.

### The microbial profile within a subject

**Fig. 2:**
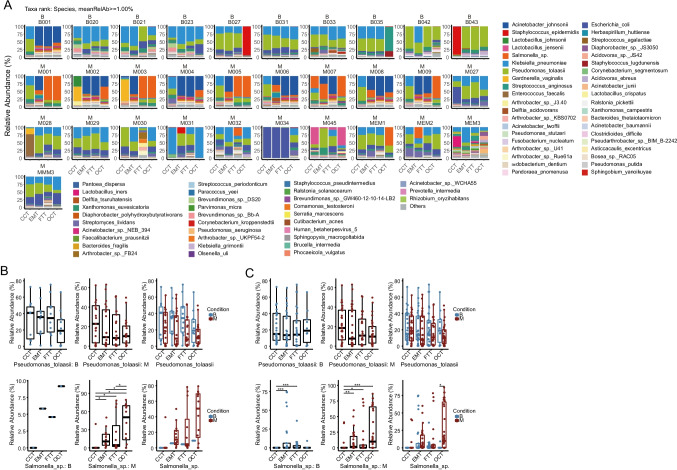
Signature species within and between individuals. **A** Species-level relative abundance community barplot analysis at 4 sites per patient. **B** The relative abundance of *Salmonella* sp. and *P. tolaasii* in benign and malignant patients presented in **A** with the relative abundance of *Salmonella* sp. more than 1% at any site (CCT, EMT, FTT, or OCT) and **C** the relative abundance of *Salmonella* sp. and *P. tolaasii* in total samples of benign and malignant patients

To exclude the effects of different microbial populations between/among subjects, we further analyzed the microbial portfolio of four different sites of single subjects. Briefly, we analyzed the relative abundance of microbes at the species level of four sites in 10 patients from control group and 24 subjects from experimental group. The samples of these 34 cases were divided into two clusters according to the *salmonella sp.* abundance (> 1%), depending on the environmental control and sample classification. We then analyzed the distribution of *Salmonella* sp. and *Pseudomonas Tolaasii* in benign and malignant patients with the relative abundance of *salmonella* sp. more than 1% at any part (CCT, EMT, FTT, or OCT) (Fig. 2A). In the control group, the relative abundance of *Pseudomonas tolaasii* gradually decreased as migrated upward from the lower reproductive tract to the ovarian tissue. There was a similar trend in the malignant samples. Meanwhile, *Salmonella* sp*.* gradually enriched among the 4 sites within the experimental group. The relative abundance in the uterine cavity was significantly higher than that of the lower reproductive tract (cervix) (Fig. 2B). Interestingly, while no difference of *Pseudomonas tolaasii* abundance was detected in all samples of both groups, however, *Salmonella* sp*.* showed an enriched status in both benign and malignant uterine cavities with higher level in the ovary of control group compared to the subjects in the experimental cohort (Fig. 2C). These findings supported the notion that microorganisms in ovarian tissues originate from the reproductive tract. Additionally, these data imply that the migration of the organisms in the upper reproductive tract of the malignant patients was different from those of benign controls with noticeable enriched microorganisms.

### Malignant ovarian tissue had distinct microbial signatures

**Fig. 3:**
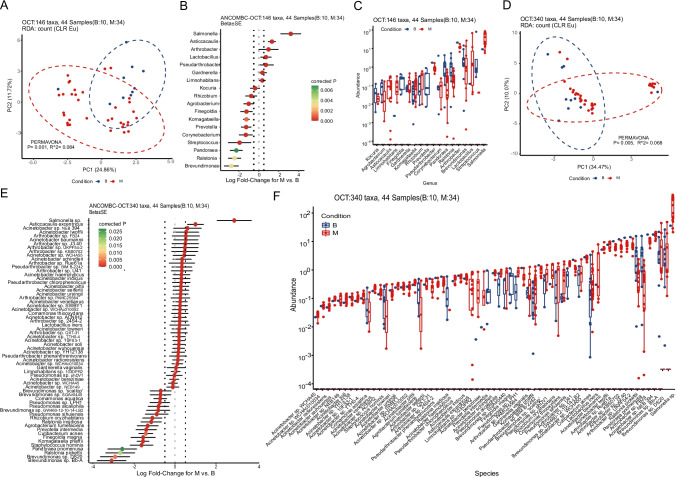
The distribution of microorganisms on the surface of benign and malignant ovarian tissues. **A** Genus-level beta-diversity analysis between 10 benign ovarian tissues and 34 malignant ones by PERMAVONA analysis (*p* = 0.001). **B** Differential abundant genus between two groups by ANCOM-BC test. C. Relative abundances of the differential abundant genus between two groups. **D**, **E**, and **F** show species’ signature

To clarify the distribution of microorganisms in malignant ovarian tissues, the ovarian samples from both groups were compared (34 subjects from experimental group and 10 subjects from control group). The two groups showed significant differences in the distribution of β-diversity (*p* = 0.001, and *p* = 0.005) at the genus level as well as the species level (Fig. 3A and D). There were 146 genus-level OTUs in all the ovarian samples. The differential bacteria enriched on the surface of malignant ovarian tissues were *Salmonella*, *Asticcacaulis*, *Arthrobacter*, *Lactobacillus*, *Pseudarthrobacter*, *and Pseudarthrobacter* compared to the top 5 different bacteria enriched on the surface of benign ovarian tissue, *Brevundimonas*, *Ralstonia*, *Pandoraea*, *Streptococcus*, *and Corynebacterium* (Fig. 3B and C). A total of 329 OTUs at the species level, *Salmonella* sp., *Asticcacaulis excentricus*, *Acinetobacter* sp. NEB 394, *Acinetobacter lwoffii*, and *Arthrobacter* sp. FB24, were enriched in malignant tissues. As for control group, *Brevundimonas* sp. Bb-A, *Brevundimonas* sp. DS20, *Ralstonia pickettii*, *Pandoraea pnomenusa*, *Staphylococcus hominis*, *Komagataella phaffii*, *Finegoldia magna*, *Cutibacterium acnes*, *Prevotella intermedia*, and *Agrobacterium tumefaciens* were enriched (Fig. 3E and F). Notably, *Salmonella* sp. was the most enriched species in malignant tissues, and its relative abundance was also the highest in the bacteria. While this study was not geared towards the detailed genotyping of every bacterium, however, *Salmonella typhimurium* is reported as one of the major species in ovarian cancer [4].

### The influence of tubal ligation on the distribution of microorganisms on the surface of ovarian cancer tissue

**Fig. 4:**
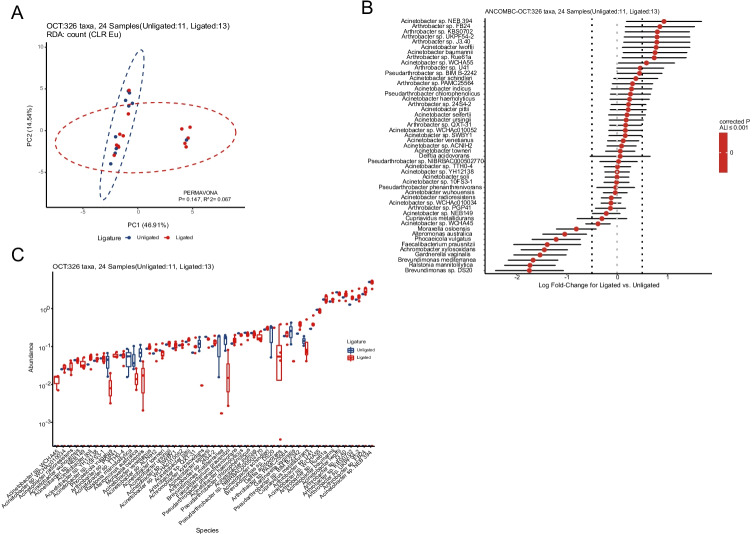
Signature species on the surface of ovarian tissues between ligation and unligation of patients with ovarian cancer. **A** Species-level beta-diversity analysis between 13 ligation and 11 unligation of patients by PERMAVONA analysis (*p* = 0.147). **B** Differential abundant species between two groups by ANCOM-BC test. **C** Relative abundances of the differential abundant species between two groups

A total of 312 OTUs were found between 13 fallopian tube ligated patients and 11 non-ligation patients. Although there was no statistical difference (*p* = 0.146), however, the β-diversity showed that the two groups share unidentical compositions of microorganisms (Fig. 4A). This might be due to the insufficient sample numbers. Among the different species, the top 5 most enriched bacteria were *Arthrobacter* sp. J3.40, *Arthrobacter* sp. UKPF54-2, *Arthrobacter* sp. KBS0702, *Arthrobacter* sp. FB24, and *Acinetobacter* sp. NEB 394 on the surface of the ovarian tissue in the ligated subjects. As for the ovarian tissue of the unligated subjects, the most enriched species were *Brevundimonas* sp. DS20, *Ralstonia mannitolilytica*, *Brevundimonas mediterranea*, *Gardnerella vaginalis*, and *Achromobacter xylosoxidans* (Fig. 4B and C).

### Platinum resistance and the microorganism distribution of the ovarian cancer tissues

**Fig. 5:**
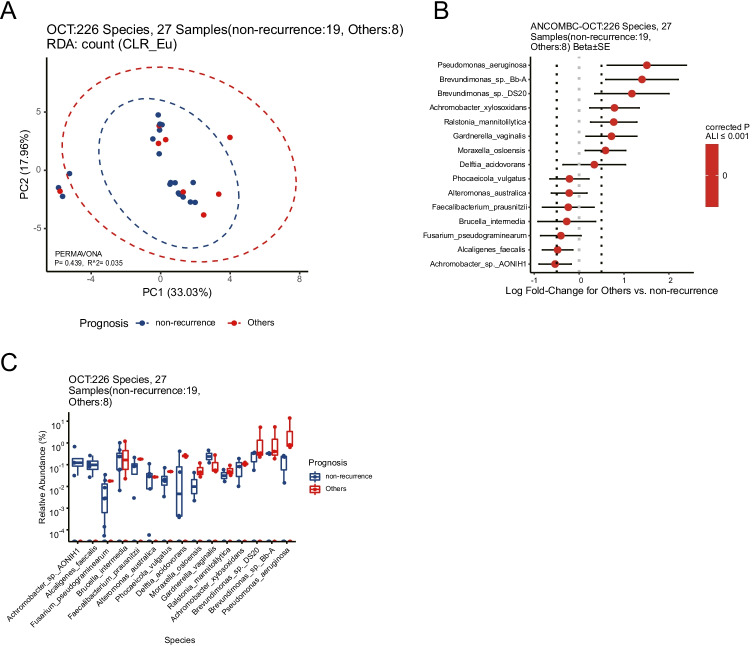
Signature species on the surface of ovarian tissues between nineteen postoperative platinum-sensitive and eight platinum-resistant patients. **A** Species-level beta-diversity analysis indicated no difference in species between the two groups. **B** Differential abundant species between two groups by ANCOMBC test. **C** Relative abundances of the differential abundant species between two groups

We compared the ovarian tissue surface microbial species between 19 postoperative platinum-sensitive and eight platinum-resistant patients. On the species level, the two groups shared 226 species (Fig. 5A). The β-diversity analysis indicated no difference in species between the two groups (*p* = 0.44). The top 5 enrichment in the platinum-resistant group is *Pseudomonas_aeruginosa*, *Ralstonia mannitolilytica*, *Achromobacter xylosoxidans*, *Brevundimonas* sp. DS20, and *Brevundimonas* sp. Bb-A (Fig. 5B and C).

## Discussion

In this work, we did the metagenomic analysis using samples from subjects with ovarian cancer that migrated from the lower reproductive tract (cervix) to the upper reproductive tract (uterine cavity, fallopian tube, and ovary). The results suggested that bacteria are the most dominant microorganism in samples within and among the groups. The relative abundance of fungi, viruses, archaea, and protists accounted for about 1%. In terms of bacteria, there was a distinct low-biomass microbiota in the upper reproductive tract of malignant ovarian cancer. There was also characteristic microbiota distribution on ovarian tissues compared with the control group. Meanwhile, there were other varieties among the existing colonies based on clinical information as well as fallopian tube ligation and platinum resistance.

There have been many studies on the correlation between local carcinogenesis and dysbiotic microbiota, pathobionts, and/or pathogens in the upper reproductive tract [11, 26–28]. Importantly, microbes enriched in tumor pose unexpected effects on the therapeutic effect of different cancer treatments (including radiotherapy, chemotherapy, and especially immunotherapy) [29, 30]. Our previous 16S rRNA sequencing analysis suggested that there were distinct microbial signatures in ovarian cancer tissue as compared to the normal fallopian tube tissue [6]. The genomic analysis could be utilized as a biologic powerhouse to tailor a preventive and most importantly an individualized strategy against numerous diseases specifically ovarian cancer in this case for our study. Additionally, the genomic analysis not only could provide accurate and more effective therapeutic and preventive approaches in ovarian cancers, but it also would facilitate the therapeutic modality in a cost-effective and less invasive fashion. Therefore, incorporation of cutting-edge metagenomic analysis similar to what was done in this study would be an excellent resource for the implication of a PPPM-based strategy to achieve a higher standard of treatment with improved level of life quality.

## Origination and association of ovarian microbiota: the footprint of PPPM

In addition to a large number of microorganisms that migrate from the lower reproductive tract, our sequencing data demonstrated that the uterine cavity locating the upper reproductive tract had a large number of microbial populations that are different from the lower reproductive tract. It is shown that microorganisms in the upper reproductive tract of benign and malignant patients mainly come from the lower reproductive tract [13]. The abdominal environment and the systemic circulatory system may also influence the microbiome of the upper reproductive tract [31]. While the upper reproductive tract and the lower reproductive tract are linked directly, the pathogens in the lower reproductive tract, such as the vagina, may migrate to the upper reproductive tract and even the pelvic cavity along with the tissue structure [32]. Therefore, it is plausible to propose a scenario in which through a well-tailored “personalized” modality, specific and desired microorganisms could be populated and be colonized in the uterine cavity through the systemic circulation and then gradually move upward to the fallopian tubes and ovaries. Interestingly, we also did find a few unique taxa that were not derived from the reproductive tract on the surface of the ovary. Some previous studies reported that intestinal microbes could enter the pelvic cavity under certain conditions (intestinal permeability change) and then colonize the ovarian tissue [27, 33]. The intestinal microbiota could also colonize the lower reproductive tract via the anus and vagina and subsequently affect the upper reproductive tract [34]. Similarly, blood circulation had been reported as an independent way for microorganisms to accumulate in ovarian tissue. Collectively, since the primary source of microorganisms is the digestive tract, a specific group of microorganisms could be delivered and/or promoted through a well-defined and executed personalized process so the host could be benefitted by their protective impact as well as predictive potential to reduce morbidity in a least non-invasive fashion in a clear manifestation of PPPM strategy.

Furthermore, our study found that the bacteria enrichment on the ovarian surface of the ligated group differed from that of the non-ligated subject but with no significant statistical differences which could be due to limited sample size. Several studies have reported that tubal ligation can cut off the path of upper reproductive tract gynecological microorganisms to the ovary, suggesting that ligation could reduce the recurrence of ovarian cancer [35]. This could be well due to the possibility that fallopian tube ligation could change the blood supply of the ovaries [9] or simply because ligation could cut off the structural path of all possible pathogenic substances from the vagina.

Compared with benign lesions, the local microenvironment of ovarian cancer did display an obvious microbial signature. Although it is already reported that host’s response to local microbial dysbiosis was crucial to the study of tumorigenesis [36], however, whether the cancer-associated microbiota leads to tumor-permissive microenvironment is still unclear. This study suggested that there are enriched pathogens, *Salmonella*, on the surface of multiple ovarian cancer tissues. Previous researches have reported that patients with Salmonella infection were six times more likely to suffer hepatobiliary cancer. *Salmonella* may participate in many pathogenic reactions that promote the occurrence and progress of cancer [37]. In addition, we also observed a higher abundance of *Brevundimonas* sp. in the control group.

Overall, regardless the causative mechanism, the genomic analysis similar to what was done in this study could identify the beneficial microorganism in the host and provide a prescription for populating the microorganisms based on personalized needs in a PPPM paradigm. In this way, the alteration of microbial portfolio would affect host microenvironment which would be affecting homeostasis, suggesting a significant clinical value to genomic analysis.

## Microorganisms associated with treatment outcomes

Specific microbial colonies may enhance the therapeutic effect during the adjuvant treatment of tumors [14, 15, 38]. Studies of intestinal microbiome on affecting the effectiveness of chemotherapy drugs, enhancing the response to radiotherapy, and regulating the immune response of the body have been reported successively [39, 40]. The recent advances in the understanding of innate immunity and microbiota have ushered in a new era in the continued efforts to better understand the treatment and prevention of carcinoma [41].

However, evidence regarding the tumor local microbiota and their function in manipulating radiotherapy, chemotherapy, and immunotherapy is still unavailable. On the other hand, although the treatment of ovarian cancer has greatly improved over the last 50 years, the options for clinicians still limit in integrating optimal surgery and systemic therapy (chemotherapy, targeted therapy, etc.). Thus, the implication of PPPM in ovarian cancer is emerging not only as a cutting-edge approach in the process of treatment, but also as an effective way in the total cancer care. The identification of predictive biomarkers and the explication of mechanism of the recurrence and resistance are the cornerstone to further improve treatment effectiveness and to develop new generation of microbio-bio-treatment [42–44]. In this study, we found enriched differential colonies on the surface of ovarian cancer tissues in the platinum-resistant group, which indicated that these bacterial groups may be the potential targets for improving ovarian cancer therapies after drug resistance. Moreover, the understanding of the critical interactions between microbe and the host would help researchers utilizing patient responsiveness in the improvement of future therapeutic agents. Importantly, the identification of a site-specific microbiome may benefit the prediction and prevention of different types of ovarian cancer and it may even strengthen the therapeutic effect of the adjuvant treatment of cancer [45]. Elucidating these complex host–microbiome interactions, including the changes from lower reproductive tract to the upper reproductive tract, will translate into interventions for prevention, diagnosis, and therapeutic effects in a personalized fashion, enhancing health outcomes for women with ovary cancer.

Finally, several reports have indicated that certain diseases and conditions including, not limited to, cancer have specific microbiome signature [31, 46]. The cancer-associated alteration of microbiome is termed as “oncobiosis” playing a role during the initiation and progression of malignancies [31]. Numerous studies have proposed several mechanistic scenarios by which oncobiosis and the interactions between microbiome and tumors may affect the cancer progression and tumor microenvironment [31]. Alteration of microbiome may affect the way immune components are functioning within and against the tumor. Modulation of immune checkpoints and changes in the ratio of active effector immune cells versus immuno-regulatory cells are some of the examples of how microbiome can affect the tumor progression and survival. At metabolic as well as cellular levels and histology, changes in microbiome can affect the tumors by regulating oxidative stress, enhancing DNA damage, and regulating the epithelial-to-mesenchymal transition conversion, key components in cancer development [31].

## Limitations

Despite the introduction of several approaches in the treatment of ovarian cancer with a PPPM perspective, there were few limitations which should be counted and considered during the interpretation of our findings. The small sample size and the low microbial abundance of the upper reproductive tract were among the major limitations. In addition, in light of COVID-19 pandemic, some patients encountered irregularity in their postoperative treatment. Furthermore, this study lacked the consideration of the potential effect of cyclic hormones on the local microecology of ovarian cancer. Moreover, the control group was selected as patients with benign disease, which may be slightly differed from the definition of healthy control. Similarly, there was no clinical data on the long-term intake of oral contraceptives, so the microbiological composition of subjects using the oral contraceptive was not analyzed. Additionally, having the phynotypic and genotypic analysis of several members of microbiome would have been highly informative, granting future investigations.

## Conclusions and expert recommendations

We live in a constant symbiosis with thousands of distinct bacterial strains that have co-evolved with us. Besides the oral-digestive and respiratory tracts, the reproductive system is also a major microbial habitat. It is of great significance to study the microbial distribution characteristics of the reproductive tract for a comprehensive understanding of the occurrence of reproductive tract diseases and for a precise development of new therapeutic modalities. Most importantly, our findings suggest a scenario in which the host native powerhouse of microbiota may be used in a PPPM paradigm, providing a novel and cutting-edge way of treating specifically ovarian cancer and other malignancies in general. One of the major advantages of PPPM is the fact that it is highly adaptable to the advanced technologies in biomedical sciences with high performance. Therefore, the PPPM would be a great vehicle to carry the production of metagenomic analysis to promote and improve diagnosis and prognosis as well as prediction values in ovarian cancer. Envisioning a personalized medicine approach in which by categorizing the patients based on their genomes and molecular profile would revolutionize the conventional therapeutic modalities in a most effective, inexpensive, and less invasive fashion fulfilling the mission of PPPM approach.

## Supplementary Information

Below is the link to the electronic supplementary material.

ESM 1 A. Q30 ratio vs. average reads read length. B. Box diagram of number of reads samples from different parts. C. The proportion of reads with classified information in the samples from different sites. D. Species-level alpha-diversity analysis. E. Phylum-level microbial composition. (PDF 1425 KB)

Different microbiota in common (≥75% of samples) microbial composition in phylum-level adjacent locations. A. The phylum-level microbiota between CCT and EMT. B. The relative abundance of differential phyla on all samples between CCT and EMT by the Wilcox-rank test. C and D. The phylum-level microbiota between EMT and FTT; E and F: The phylum-level microbiota between FTT and OCT. (PDF 2175 KB)

Different microbiota in common (≥75% of samples) microbial composition in species-level adjacent locations. A. The species-level microbiota between CCT and EMT. B. The relative abundance of differential species on all samples in between CCT and EMT by Wilcox-rank test. C and D. The species-level microbiota between EMT and FTT; E and F: The species-level microbiota between FTT and OCT. (PDF 6378 KB)

## Data Availability

Not applicable.

## Code availability

The analyses methods and used packages are illustrated in the “Materials and methods” section. All other R code and analyses are available from the corresponding author upon request.

## References

[1] Torre LA, Trabert B, DeSantis CE, Miller KD, Samimi G, Runowicz CD (2018). Ovarian cancer statistics, 2018. CA Cancer J Clin..

[2] Siegel RL, Miller KD, Fuchs HE, Jemal A (2021). Cancer statistics, 2021. CA Cancer J Clin..

[3] Fernandez-Garza LE, Dominguez-Vigil IG, Garza-Martinez J, Valdez-Aparicio EA, Barrera-Barrera SA, Barrera-Saldana HA (2021). Personalized medicine in ovarian cancer: a perspective from Mexico. World J Oncol..

[4] Matsumoto Y, Miwa S, Zhang Y, Zhao M, Yano S, Uehara F (2015). Intraperitoneal administration of tumor-targeting Salmonella typhimurium A1-R inhibits disseminated human ovarian cancer and extends survival in nude mice. Oncotarget..

[5] Banerjee S, Tian T, Wei Z, Shih N, Feldman MD, Alwine JC (2017). The ovarian cancer oncobiome. Oncotarget..

[6] Zhou B, Sun C, Huang J, Xia M, Guo E, Li N (2019). The biodiversity composition of microbiome in ovarian carcinoma patients. Sci Rep..

[7] Chen L, Zhai Y, Wang Y, Fearon ER, Nunez G, Inohara N (2021). Altering the microbiome inhibits tumorigenesis in a mouse model of oviductal high-grade serous carcinoma. Cancer Res..

[8] Janavicius R (2010). Founder BRCA1/2 mutations in the Europe: implications for hereditary breast-ovarian cancer prevention and control. EPMA J..

[9] Rice MS, Hankinson SE, Tworoger SS (2014). Tubal ligation, hysterectomy, unilateral oophorectomy, and risk of ovarian cancer in the Nurses’ Health Studies. Fertil Steril..

[10] Nejman D, Livyatan I, Fuks G, Gavert N, Zwang Y, Geller LT (2020). The human tumor microbiome is composed of tumor type-specific intracellular bacteria. Science..

[11] Walther-Antonio MR, Chen J, Multinu F, Hokenstad A, Distad TJ, Cheek EH (2016). Potential contribution of the uterine microbiome in the development of endometrial cancer. Genome Med..

[12] Chen C, Song X, Wei W, Zhong H, Dai J, Lan Z (2017). The microbiota continuum along the female reproductive tract and its relation to uterine-related diseases. Nat Commun..

[13] Laniewski P, Ilhan ZE, Herbst-Kralovetz MM (2020). The microbiome and gynaecological cancer development, prevention and therapy. Nat Rev Urol..

[14] Routy B, Le Chatelier E, Derosa L, Duong CPM, Alou MT, Daillere R (2018). Gut microbiome influences efficacy of PD-1-based immunotherapy against epithelial tumors. Science..

[15] Matson V, Fessler J, Bao R, Chongsuwat T, Zha Y, Alegre ML (2018). The commensal microbiome is associated with anti-PD-1 efficacy in metastatic melanoma patients. Science..

[16] Yang B, Fan J, Huang J, Guo E, Fu Y, Liu S (2021). Clinical and molecular characteristics of COVID-19 patients with persistent SARS-CoV-2 infection. Nat Commun..

[17] Chen S, Zhou Y, Chen Y, Gu J (2018). fastp: an ultra-fast all-in-one FASTQ preprocessor. Bioinformatics..

[18] Langmead B, Salzberg SL (2012). Fast gapped-read alignment with Bowtie 2. Nat Methods..

[19] Li H, Handsaker B, Wysoker A, Fennell T, Ruan J, Homer N (2009). The sequence alignment/map format and SAMtools. Bioinformatics..

[20] Wood DE, Lu J, Langmead B (2019). Improved metagenomic analysis with Kraken 2. Genome Biol..

[21] Unterseher M, Jumpponen A, Opik M, Tedersoo L, Moora M, Dormann CF (2011). Species abundance distributions and richness estimations in fungal metagenomics–lessons learned from community ecology. Mol Ecol..

[22] McMurdie PJ, Holmes S (2013). phyloseq: an R package for reproducible interactive analysis and graphics of microbiome census data. PLoS One..

[23] Knight R, Vrbanac A, Taylor BC, Aksenov A, Callewaert C, Debelius J (2018). Best practices for analysing microbiomes. Nat Rev Microbiol..

[24] Tang ZZ, Chen G, Alekseyenko AV (2016). PERMANOVA-S: association test for microbial community composition that accommodates confounders and multiple distances. Bioinformatics..

[25] Davis A, Tinker AV, Friedlander M (2014). “Platinum resistant” ovarian cancer: what is it, who to treat and how to measure benefit?. Gynecol Oncol..

[26] Chen J, Domingue JC, Sears CL (2017). Microbiota dysbiosis in select human cancers: evidence of association and causality. Semin Immunol..

[27] Chase D, Goulder A, Zenhausern F, Monk B, Herbst-Kralovetz M (2015). The vaginal and gastrointestinal microbiomes in gynecologic cancers: a review of applications in etiology, symptoms and treatment. Gynecol Oncol..

[28] Shanmughapriya S, Senthilkumar G, Vinodhini K, Das BC, Vasanthi N, Natarajaseenivasan K (2012). Viral and bacterial aetiologies of epithelial ovarian cancer. Eur J Clin Microbiol Infect Dis..

[29] Seong E, Bose S, Han SY, Song EJ, Lee M, Nam YD (2021). Positive influence of gut microbiota on the effects of Korean red ginseng in metabolic syndrome: a randomized, double-blind, placebo-controlled clinical trial. EPMA J..

[30] Kudela E, Liskova A, Samec M, Koklesova L, Holubekova V, Rokos T (2021). The interplay between the vaginal microbiome and innate immunity in the focus of predictive, preventive, and personalized medical approach to combat HPV-induced cervical cancer. EPMA J..

[31] Sipos A, Ujlaki G, Miko E, Maka E, Szabo J, Uray K (2021). The role of the microbiome in ovarian cancer: mechanistic insights into oncobiosis and to bacterial metabolite signaling. Mol Med..

[32] Xu J, Peng JJ, Yang W, Fu K, Zhang Y (2020). Vaginal microbiomes and ovarian cancer: a review. Am J Cancer Res..

[33] Zhang J, Liu M, Ke S, Huang X, Fang S, He M (2021). Gut and vagina microbiota associated with estrus return of weaning sows and its correlation with the changes in serum metabolites. Front Microbiol..

[34] Thomas-White K, Forster SC, Kumar N, Van Kuiken M, Putonti C, Stares MD (2018). Culturing of female bladder bacteria reveals an interconnected urogenital microbiota. Nat Commun..

[35] Falconer H, Yin L, Salehi S, Altman D (2021). Association between pelvic inflammatory disease and subsequent salpingectomy on the risk for ovarian cancer. Eur J Cancer..

[36] Sepich-Poore G D, Zitvogel L, Straussman R, Hasty J, Wargo J A,Knight R. The microbiome and human cancer. Science. 2021; 371 (6536). 10.1126/science.abc4552 10.1126/science.abc4552 PMC8767999 33766858

[37] Welton JC, Marr JS, Friedman SM (1979). Association between hepatobiliary cancer and typhoid carrier state. Lancet..

[38] Sims TT, El Alam MB, Karpinets TV, Dorta-Estremera S, Hegde VL, Nookala S (2021). Gut microbiome diversity is an independent predictor of survival in cervical cancer patients receiving chemoradiation. Commun Biol..

[39] Alexander JL, Wilson ID, Teare J, Marchesi JR, Nicholson JK, Kinross JM (2017). Gut microbiota modulation of chemotherapy efficacy and toxicity. Nat Rev Gastroenterol Hepatol..

[40] Vetizou M, Pitt JM, Daillere R, Lepage P, Waldschmitt N, Flament C (2015). Anticancer immunotherapy by CTLA-4 blockade relies on the gut microbiota. Science..

[41] Yu JC, Khodadadi H, Baban B (2019). Innate immunity and oral microbiome: a personalized, predictive, and preventive approach to the management of oral diseases. EPMA J..

[42] Lheureux S, Braunstein M, Oza AM (2019). Epithelial ovarian cancer: evolution of management in the era of precision medicine. CA Cancer J Clin..

[43] Sun C, Yin J, Fang Y, Chen J, Jeong KJ, Chen X (2018). BRD4 inhibition is synthetic lethal with PARP inhibitors through the induction of homologous recombination deficiency. Cancer Cell..

[44] Paltsev M, Kiselev V, Muyzhnek E, Drukh V, Kuznetsov I, Pchelintseva O (2013). Comparative preclinical pharmacokinetics study of 3,3'-diindolylmethane formulations: is personalized treatment and targeted chemoprevention in the horizon?. EPMA J..

[45] Li N, Zhan X (2020). Anti-parasite drug ivermectin can suppress ovarian cancer by regulating lncRNA-EIF4A3-mRNA axes. EPMA J..

[46] Hodson R (2021). Ovarian cancer. Nature..

