# Supplementary figures and images for: Metagenomic analysis of the microbiome of the upper reproductive tract: combating ovarian cancer through predictive, preventive, and personalized medicine
Source: EPMA J. 2022 Jun 23;13(3):487–98. doi: 10.1007/s13167-022-00286-1 (PMC9219379; doi:10.1007/s13167-022-00286-1)

A

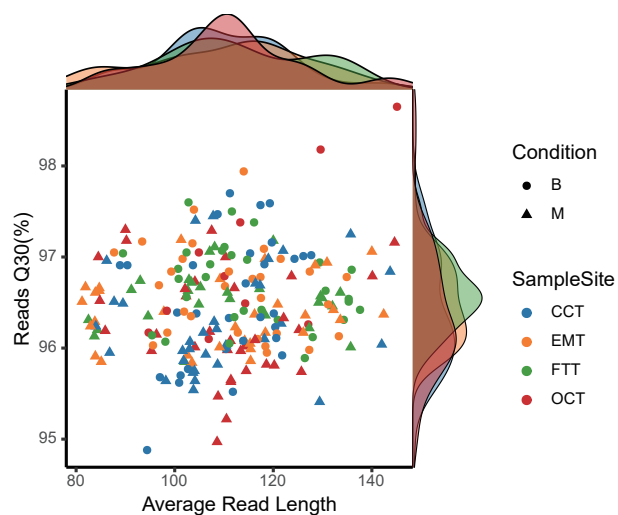

B

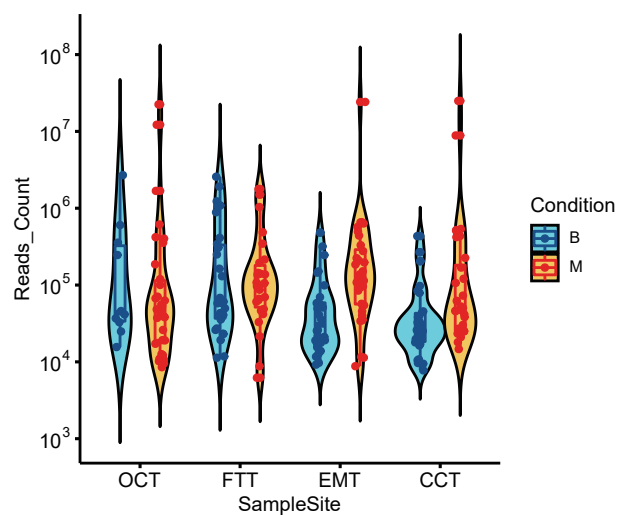

C

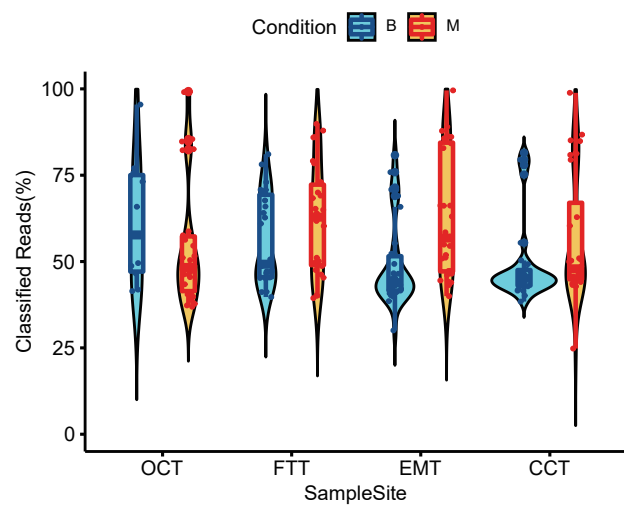

D

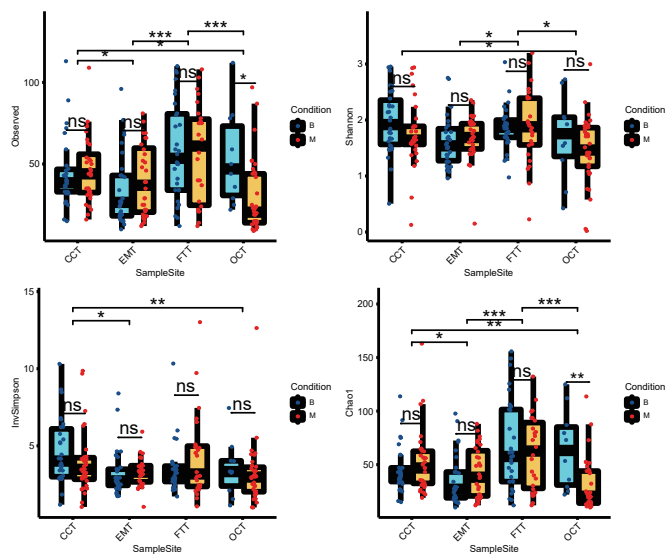

E

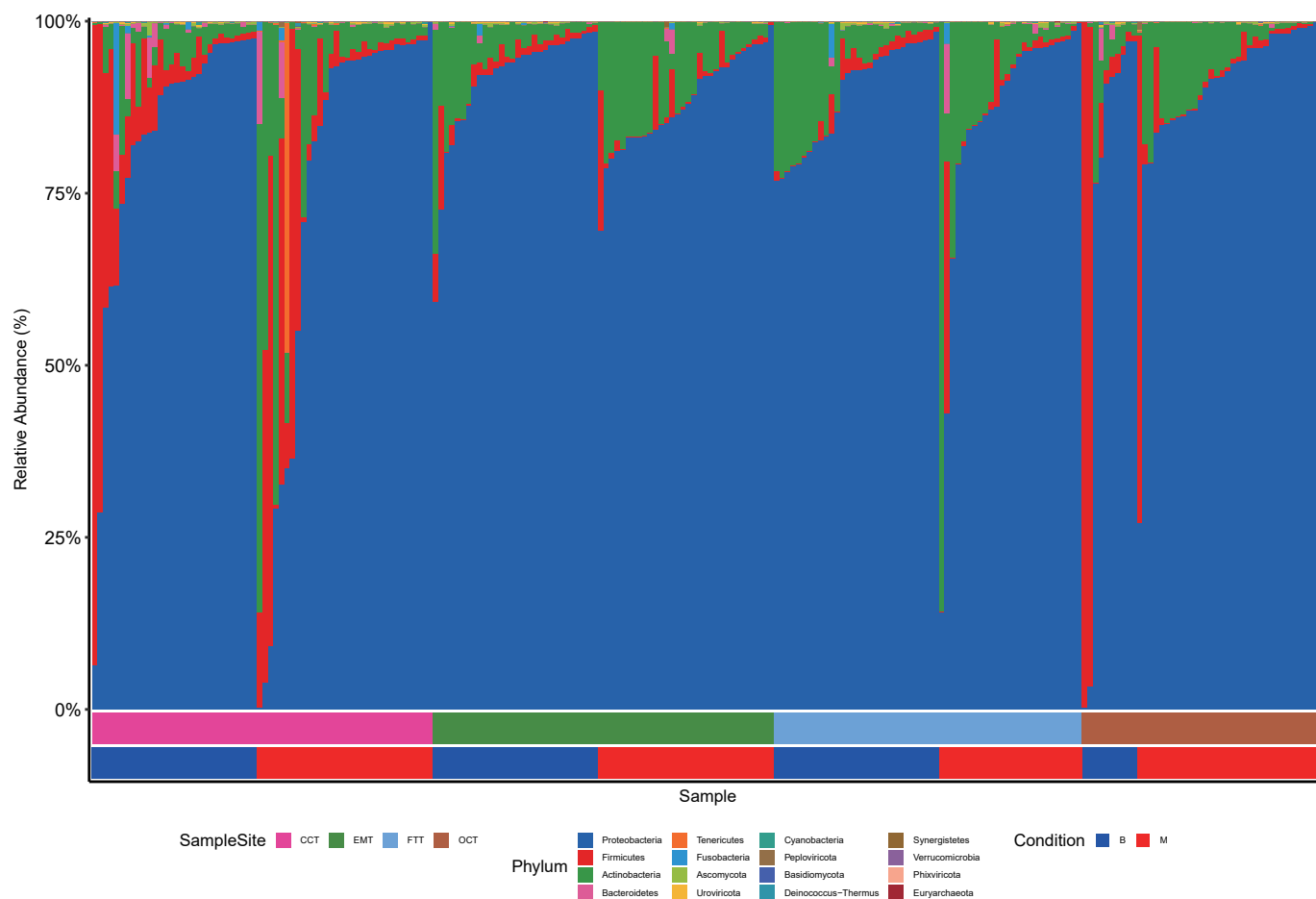

Supplement: Supplementary file 1 — A. Q30 ratio vs. average reads read length. B. Box diagram of number of reads samples from different parts. C. The proportion of reads with classified information in the samples from different sites. D. Species-level alpha-diversity analysis. E. Phylum-level microbial composition. (PDF 1425 KB) [file 13167_2022_286_MOESM1_ESM.pdf]

A

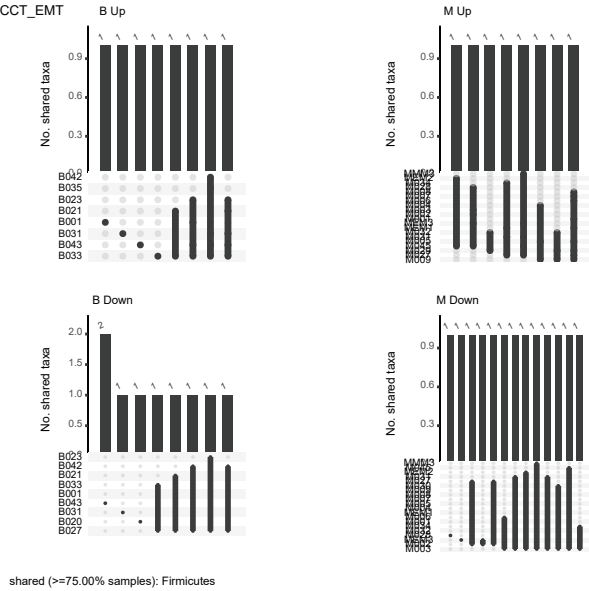

C

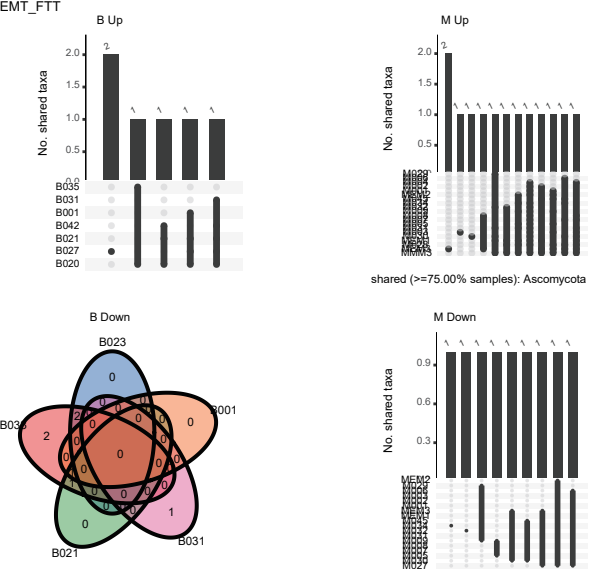

E

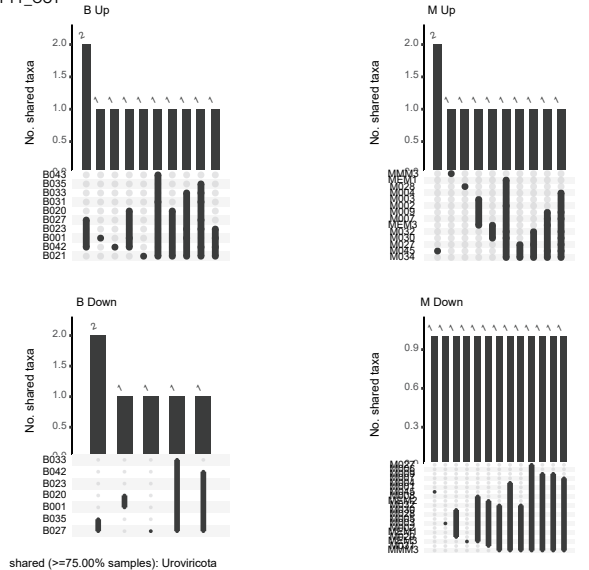

B

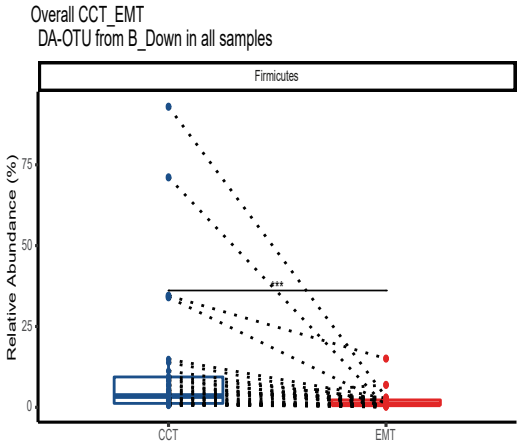

D

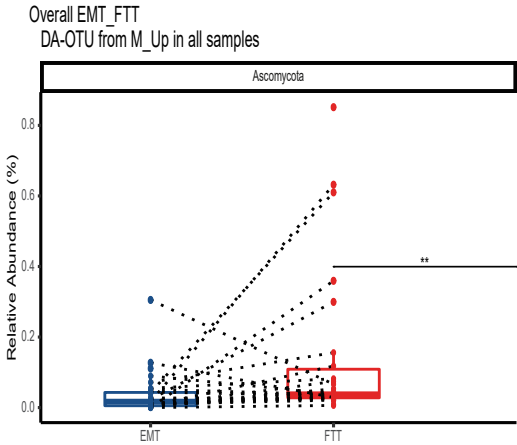

F

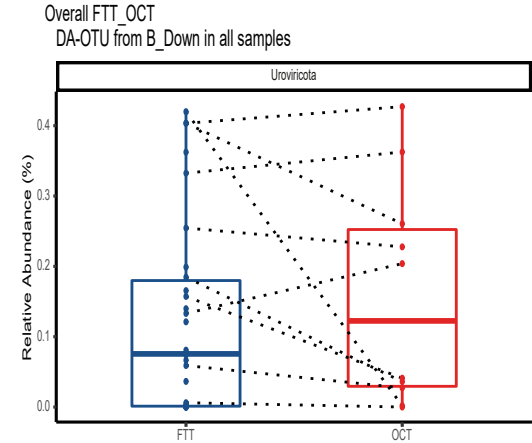

Supplement: Supplementary file 2 — Different microbiota in common (≥75% of samples) microbial composition in phylum-level adjacent locations. A. The phylum-level microbiota between CCT and EMT. B. The relative abundance of differential phyla on all samples between CCT and EMT by the Wilcox-rank test. C and D. The phylum-level microbiota between EMT and FTT; E and F: The phylum-level microbiota between FTT and OCT. (PDF 2175 KB) [file 13167_2022_286_MOESM2_ESM.pdf]

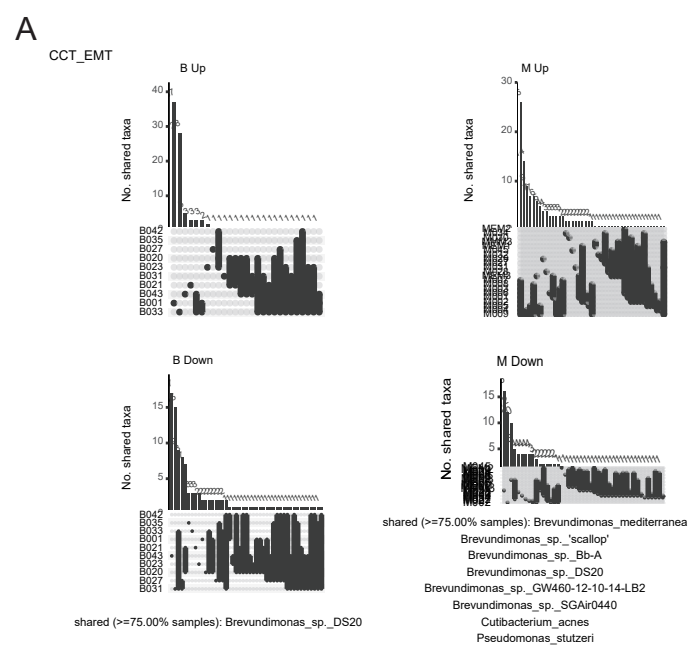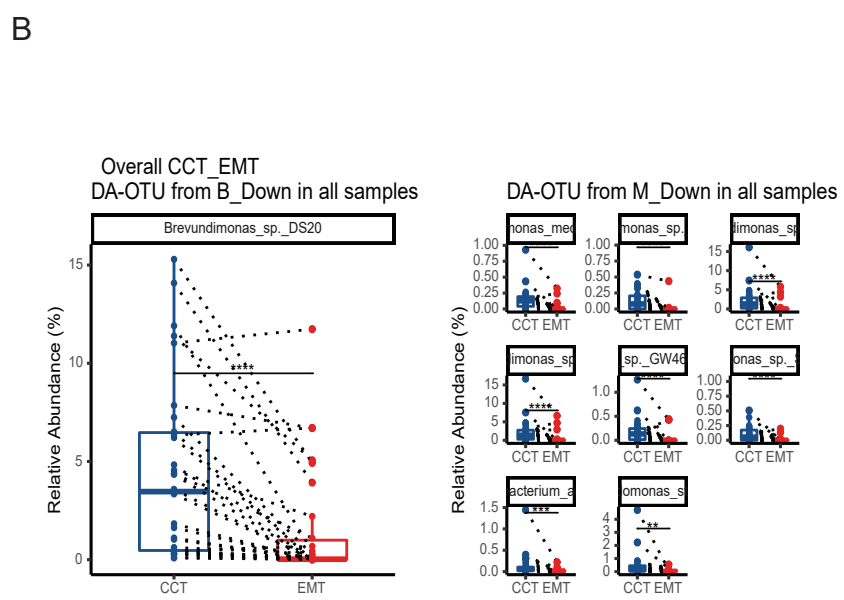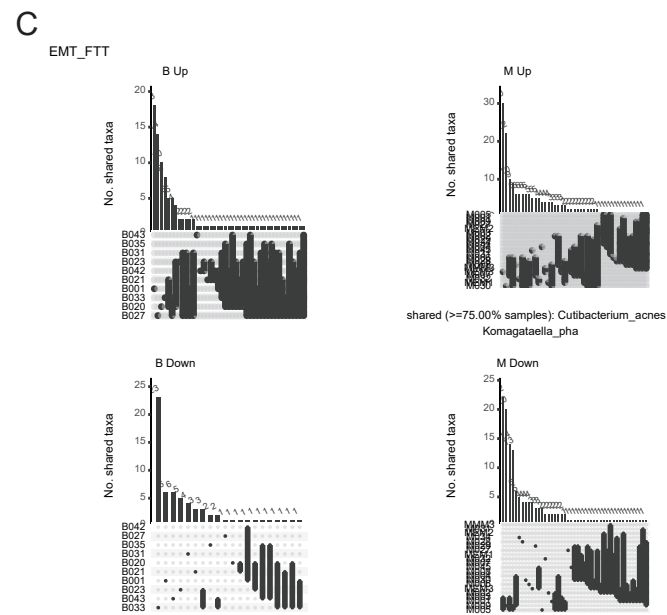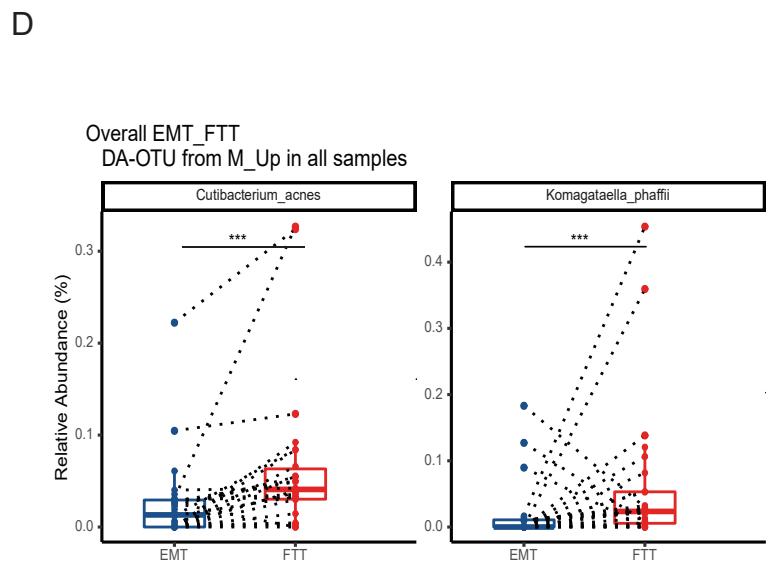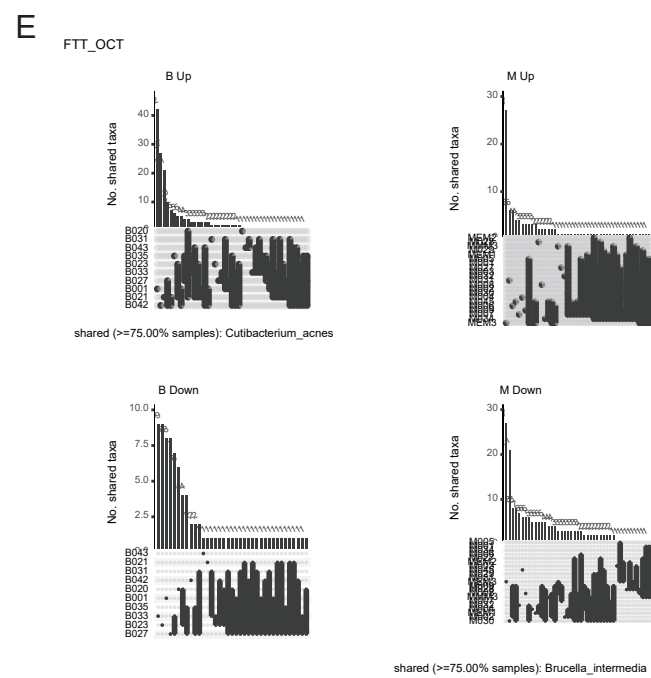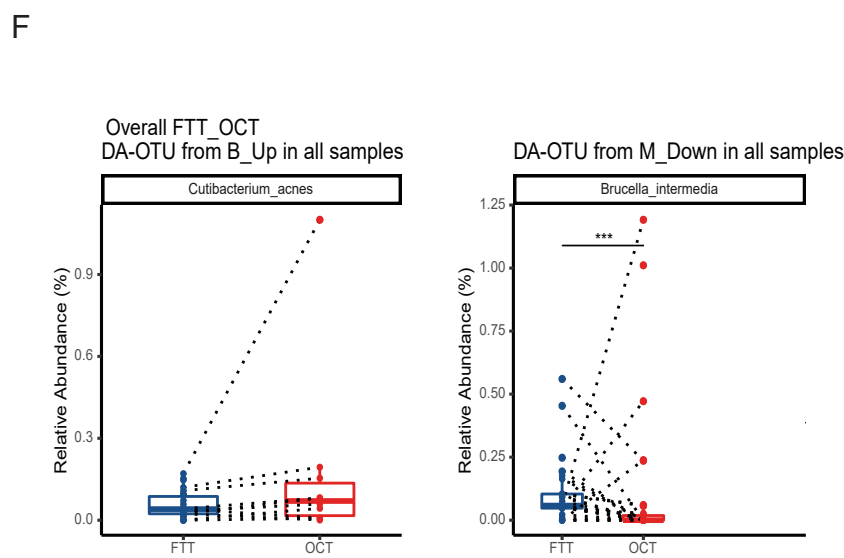

Supplement: Supplementary file 3 — Different microbiota in common (≥75% of samples) microbial composition in species-level adjacent locations. A. The species-level microbiota between CCT and EMT. B. The relative abundance of differential species on all samples in between CCT and EMT by Wilcox-rank test. C and D. The species-level microbiota between EMT and FTT; E and F: The species-level microbiota between FTT and OCT. (PDF 6378 KB) [file 13167_2022_286_MOESM3_ESM.pdf]
